# Biologization of Pcl-Mesh Using Platelet Rich Fibrin (Prf) Enhances Its Regenerative Potential In Vitro

**DOI:** 10.3390/ijms22042159

**Published:** 2021-02-22

**Authors:** Sarah Al-Maawi, Eva Dohle, Jing Lim, Paul Weigl, Swee Hin Teoh, Robert Sader, Shahram Ghanaati

**Affiliations:** 1 FORM, Frankfurt Oral Regenerative Medicine, Clinic for Maxillofacial and Plastic Surgery, Goethe University, Theodor-Stern-Kai 7, 60596 Frankfurt, Germany; sarah.al-maawi@kgu.de (S.A.-M.); Eva.Dohle@kgu.de (E.D.); R.Sader@em.uni-frankfurt.de (R.S.); 2 Osteopore International, Singapore 618305, Singapore; Lim_Jing@osteopore.com; 3 Department of Prosthodontics and Head of Department of Postgraduate Education, Center for Dentistry and Oral Medicine (Carolinum), Goethe University, Theodor-Stern-Kai 7, 60596 Frankfurt, Germany; weigl@em.uni-frankfurt.de; 4 School of Chemical and Biomedical Engineering/Lee Kong Chian School of Medicine, Nanyang Technological University, Singapore 637459, Singapore; teohsh@ntu.edu.sg

**Keywords:** platelet-rich-fibrin, bone-regeneration, biologization, regenerative therapy, blood concentrates, LSCC

## Abstract

Introduction: Resorbable synthetic scaffolds are promising for different indications, especially in the context of bone regeneration. However, they require additional biological components to enhance their osteogenic potential. In addition to different cell types, autologous blood-derived matrices offer many advantages to enhance the regenerative capacity of biomaterials. The present study aimed to analyze whether biologization of a PCL-mesh coated using differently centrifuged Platelet rich fibrin (PRF) matrices will have a positive influence on primary human osteoblasts activity in vitro. A polymeric resorbable scaffold (Osteomesh, Osteopore^TM^ (OP), Singapore) was combined with differently centrifuged PRF matrices to evaluate the additional influence of this biologization concept on bone regeneration in vitro. Peripheral blood of three healthy donors was used to gain PRF matrices centrifuged either at High (710× *g*, 8 min) or Low (44× *g*, 8 min) relative centrifugal force (RCF) according to the low speed centrifugation concept (LSCC). OP-PRF constructs were cultured with pOBs. POBs cultured on the uncoated OP served as a control. After three and seven days of cultivation, cell culture supernatants were collected to analyze the pOBs activity by determining the concentrations of VEGF, TGF-β1, PDGF, OPG, IL-8, and ALP- activity. Immunofluorescence staining was used to evaluate the Osteopontin expression of pOBs. After three days, the group of OP+PRF_Low_+pOBs showed significantly higher expression of IL-8, TGF-ß1, PDGF, and VEGF compared to the group of OP+PRF_High_+pOBs and OP+pOBs. Similar results were observed on day 7. Moreover, OP+PRF_Low_+pOBs exhibited significantly higher activity of ALP compared to OP+PRF_High_+pOBs and OP+pOBs. Immunofluorescence staining showed a higher number of pOBs adherent to OP+PRF_Low_+pOBs compared to the groups OP+PRF_High_+pOBs and OP+pOBs. To the best of our knowledge, this study is the first to investigate the osteoblasts activity when cultured on a PRF-coated PCL-mesh in vitro. The presented results suggest that PRF_Low_ centrifuged according to LSCC exhibits autologous blood cells and growth factors, seem to have a significant effect on osteogenesis. Thereby, the combination of OP with PRF_Low_ showed promising results to support bone regeneration. Further in vivo studies are required to verify the results and carry out potential results for clinical translation.

## 1. Introduction

Three-dimensional bone reconstruction and regeneration remains a challenging indication in oral and maxillofacial surgery. Different biomaterials were introduced to support bone augmentation, reconstruction, and regeneration. To achieve successful clinical outcomes, biomaterials have to meet several clinical requirements. In this context, mechanical stability, biocompatibility, and an enhanced regenerative capacity are highly needed [1]. In general, biomaterials are either gained from natural sources (e.g., xenogeneic, allogeneic, phycogeneic) or manufactured synthetically [2]. Naturally derived biomaterials must undergo strict purification and processing to eliminate biologically active components and reduce the risk of infection or adverse immune response [3]. Thereby, the processing of biomaterials results mostly in biologically inactive biomaterials that are mainly used to serve as a scaffold and to support tissue regeneration. Similarly, synthetic biomaterials are designed and manufactured to allow cell adhesion, proliferation and regeneration [4,5]. However, these biomaterials are biologically inactive, if not additionally loaded with specific proteins.

Although a wide variety of bioactive and osteoinductive biomaterials have been developed during preclinical research, only very few were certified to be used in clinical application such as bone morphogenetic proteins (BMP) [6,7]. Clinical trials for over a decade investigated the application of BMP loaded biomaterials for bone regeneration in different clinical indications. Some studies have shown a clinical benefit [8], especially for the regeneration of complex bone defects, others warned from serious complications when applying a high dose [9]. Most bioactive biomaterials apply high supraphysiologic concentrations. Still, the most suitable concentration for the respective indication is, to date, unknown. Additionally, the complexity of the regeneration process including many different cytokines and growth factors is meanwhile well described [10]. Therefore, some research groups focused on the development of biomaterials loaded with a combination of growth factors and cytokines and showed promising preclinical results [11]. Nevertheless, their translation to clinical applications remained unrealistic due to drawbacks such as the used dose, protein source, and lack of evidence for the most suitable combination formula. These limitations make inactive biomaterials safer and more favorable for clinical use.

Another autologous and bioactive alternative is provided by blood concentrates such as platelet-rich fibrin (PRF) [12]. PRF gained increasing importance in different clinical fields of regenerative medicine because of its natural autologous origin, its accessibility, and its promising regenerative capacity. PRF can be gained from patients own peripheral blood by single step centrifugation [13]. The resulting PRF is composed of platelets, leukocytes, and their subgroups embedded in a fibrin scaffold [14]. Many studies outlined the regenerative capacity of PRF by releasing different growth factors such as vascular endothelial growth factor (VEGF), epidermal growth factor (EGF), platelet-derived growth factor (PDGF), insulin-like growth factor (IGF), and others [15,16,17]. Recently, the low speed centrifugation concept (LSCC) was introduced to optimize the regenerative capacity of blood concentrates by influencing the applied relative centrifugal force (RCF) [18]. Thereby, it was shown that PRF-matrices that were processed using a high RCF included significantly lower number of platelets and leukocytes and released significantly lower concentrations of different growth factors compared to PRF matrices that were prepared using a low RCF [18,19,20]. Moreover, an in vitro study showed that the treatment of a co-culture combined of primary human osteoblasts and endothelial cells using PRF, prepared according to LSCC, results in formation of significantly higher number of vessel like structures compared to the untreated control [21]. Furthermore, liquid-PRF has been developed on this low-speed centrifugation concept allowing the combination of PRF prior to its conversion to fibrin with biomaterials resulting in the biological activation of these materials in a clinically easy applicable way [22,23]. The regenerative potential of liquid-PRF has been already documented in vitro [24,25] as well as in several clinical studies in medicine and dentistry strongly promoting soft tissue wound healing in the management of facial cutaneous sinus tracts or when applied in the treatment of oral lichen planus [26,27]. These findings again highlight the potential of PRF in supporting tissue regeneration.

The previously described regenerative potential of PRF makes its combination with inactive biomaterials a promising approach to biologize the biomaterial surface and enhance its regenerative capacity. Synthetic inactive biomaterials such as PCL have been successfully used in the clinic [28,29]. Its gradual degradation profile and simultaneous mechanical stability makes it a favorable candidate to support complex bone reconstruction. Therefore, the present study aimed to analyze whether biomaterial biologization using a clinically applicable PCL-mesh combined with differently centrifuged PRF-matrices might support bone regeneration in vitro. Thereby, primary human osteoblasts were cultured on either native or PRF-coated PCL-meshes with the aim to enhance their osteogenic activity and support bone regeneration. To the best of our knowledge, this study is the first to investigate the osteoblasts activity when cultured on a PRF-coated PCL-mesh.

## 2. Results

### 2.1. Prf Provides Matrix and Essential Growth Factor Supply for Human Primary Osteoblasts: Characterization of Prf Coating

**Figure 1:**
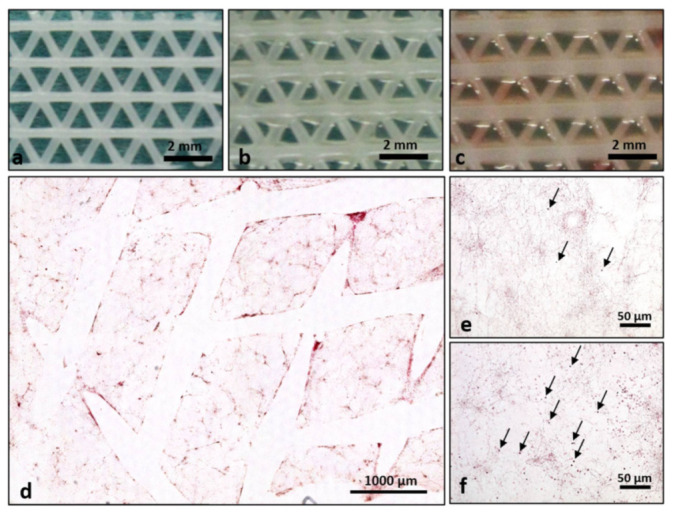
(**a**–**c**). Overview of material structure of (**a**) Osteopore alone, (**b**) Osteopore with PRF_High_ and (**c**) Osteopore with PRF_Low_. (**d**–**f**) Comparison of platelet rich fibrin (PRF) morphology of coated PCL material with high and low RCF PRF (OP+PRF_High_ and OP+PRF_Low_) without addition of primary cells. (**d**/**f**) Osteopore with PRF_Low_ and (**e**) Osteopore with PRF_High_.Black arrows point to mononuclear blood cells.

Platelet rich fibrin represents a stable three-dimensional fibrin-based matrix when added to the Osteopore^TM^ material (Figure 1b–f) compared to osteopore without PRF coating (Figure 1a). A strong fibrin network that is covering the whole material can be documented via H&E staining of histological sections when PRF was supplemented (Figure 1d–f). Comparison of cell distribution and fibrin morphology of coated material with high and low RCF PRF (OP+PRF_High_ and OP+PRF_Low_) without addition of primary cells revealed compact fibrin structure with higher amounts of cells when PRF_Low_ was added to the material (Figure 1d,f, arrows) when compared to PCL coated with PRF_high_ (Figure 1e, arrows).

**Figure 2:**
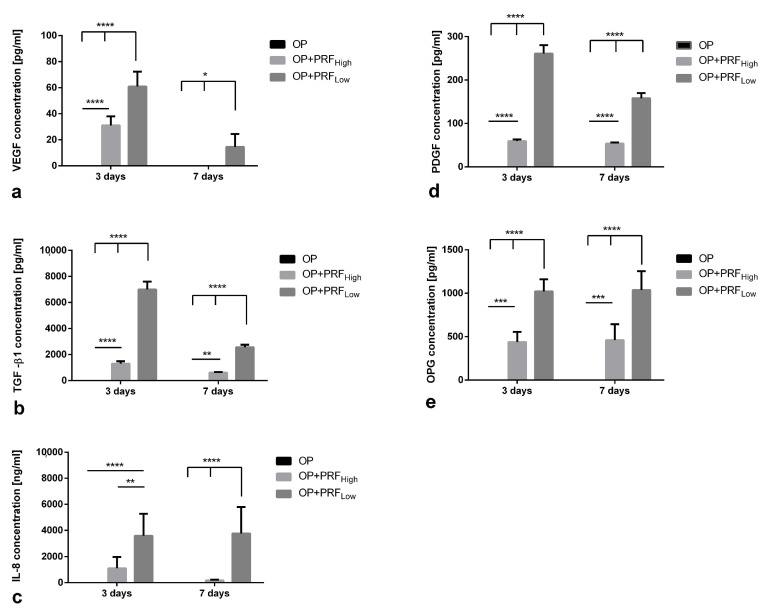
Evaluation of concentration of different growth factors and cytokines released in culture supernatants of low and high PRF coated materials (OP+PRF_High_ and OP+PRF_Low_) without primary Osteoblasts. *p*-value < 0.05 *, *p*-value < 0.01 **, *p*-value < 0.001 *** or *p*-value < 0.0001 ****.

Cell culture supernatants of low and high PRF coated materials (OP+PRF_High_ and OP+PRF_Low_) were analyzed for concentration of different growth factors and cytokines released into the surrounding cell culture medium (Figure 2). According to the morphological findings evaluated via H&E staining documenting a generally higher amount of cells in low RCF PRF (PRF_Low_), release of all analyzed growth factors and cytokines: vascular endothelial factor (VEGF), transforming growth factor β1 (TGF-β1), platelet-derived growth factor (PDGF), osteoprotegerin (OPG), and interleukin-8 (IL-8) was significantly higher in low RCF PRF (OP+PRF_Low_) supernatants after 3 and 7 days of cultivation compared to high RCF PRF (Figure 2a–e). In the control group OP (cultivation of PCL meshes without PRF), none of the estimated factors can be detected.

### 2.2. Prf Coating of Osteopore^TM^ Meshes Improves Material Biocompatibility: Adhesion and Integration of Primary Osteoblasts

**Figure 3:**
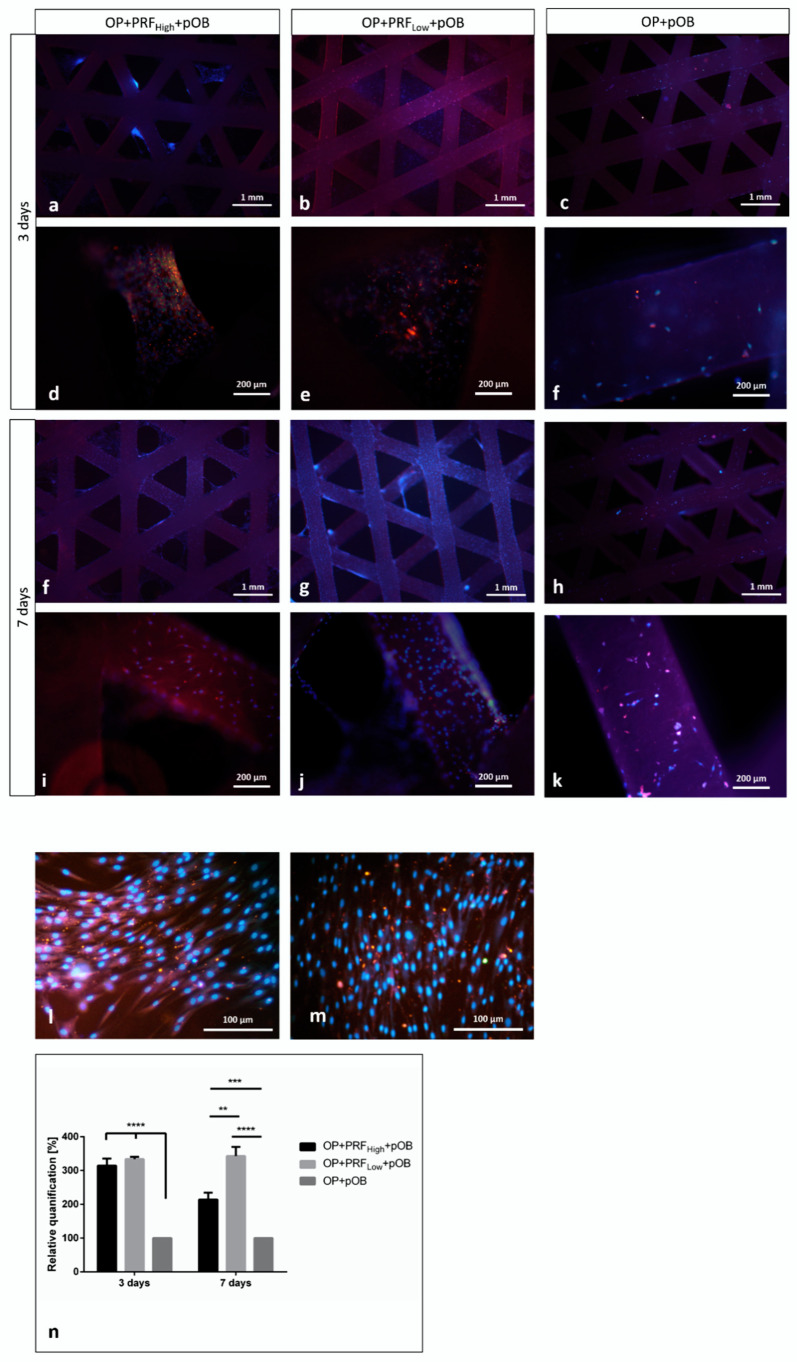
Evaluation of adhesion and integration of primary osteoblasts on PRF-coated PCL meshes. (**a**–**m**) Experimental groups (OP+PRF_High_+pOB and OP+PRF_Low_+pOB and OP+pOB) were stained immunofluorescently for osteogenic marker osteopontin as well as counterstained via Dapi. (**n**) Evaluation of alkaline phosphatase activity in the individual experimental groups. *p*-value < 0.05 *, *p*-value < 0.01 **, *p*-value < 0.001 *** or *p*-value < 0.0001 ****.

**Figure 4:**
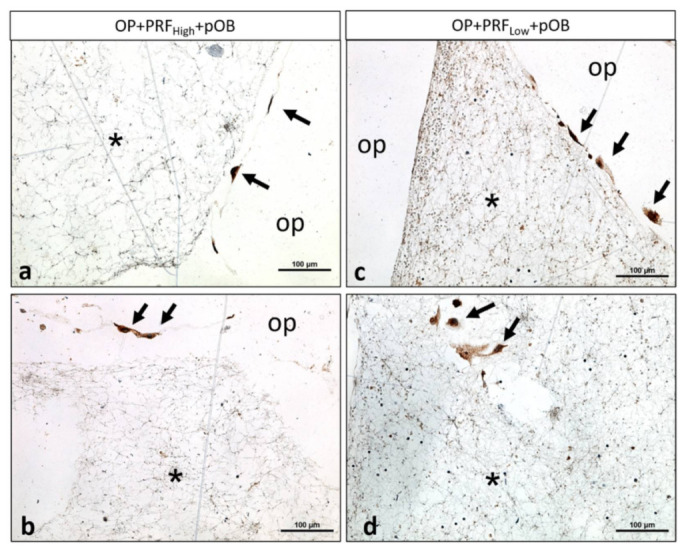
Immunohistological staining using Osteopontin shows the fibrin network (*) within the interspace of the biomaterial (OP) in both groups after seven days. (**a**,**b**) illustrate the few osteoblasts (arrows) adherent at the OP-PRF_high_ interface in the group of OP+PRF_High_+pOB compared to (**c**) the higher number of osteoblasts in the group of OP+PRF_Low_+pOB after seven days. (**d**) shows osteoblasts migrating into the fibrin network (*) that was formed within the biomaterial interspace in the group of OP+PRF_Low_+pOB.

Human primary osteoblasts were seeded on top of the Osteopore^TM^ meshes, either coated with low or high RCF PRF (OP+PRF_High_+pOB and OP+PRF_Low_+pOB) or without PRF coating (OP+pOB). Experimental groups were stained immunofluorescently for osteogenic marker osteopontin as well as counterstained via Dapi. Staining of cell nuclei using Dapi reveal the adherence of pOB and PRF with the containing blood cells on the material as well as in interspaces of the Osteopore^TM^ mesh (Figure 3a,b,d–g,i,j) when the material has been coated with PRF, independently of the applied centrifugation force used for PRF preparation and after both time points of cultivation. In contrast, when pOBs were seeded on the pure material without PRF coating, only few adherent cells can be detected on the surface of the Osteopore^TM^ meshes after seven days (Figure 3c,f,h,k). Immunofluorescent staining of primary osteoblasts using the osteoblastic specific marker osteopontin clearly illustrates osteopontin positive cells adhering on the Osteopore^TM^ material when cells were seeded on the PRF precoated Osteopore^TM^ meshes after three and seven days of cultivation (Figure 3a,b,d–g,i,j), (Figure 4a–c). In contrast, when Osteopore^TM^ is not precoated with PRF, cells adhere on the bottom of the cell culture plastic and not on the PCL after both cultivation time points, although the material was transferred to a new well 24 h after pOBs were seeded (Figure 3l,m). Determination of alkaline phosphatase enzymatic activity was used to evaluate osteoblastic functionality in response to coating of Osteopore^TM^ meshes with high and low RCF PRF compared to pOB functionality cultivated on material without coating. Evaluation of alkaline phosphatase activity in the individual experimental groups reveal a significant higher ALP activity when osteoblasts were cultivated on OP+PRF compared to pOBs cultivated on PCL alone (Figure 3n). ALP activity levels are similar in the OP+PRF groups after three days of cultivation, independently of the applied PRF. However, after seven days of cultivation highest activity of ALP can be determined when osteoblasts were cultivated on OP+PRF_Low_ compared to the other groups.

### 2.3. Stabilization of Growth Factor and Cytokine Content in Cell Culture Supernatants via Prf Coating

**Figure 5:**
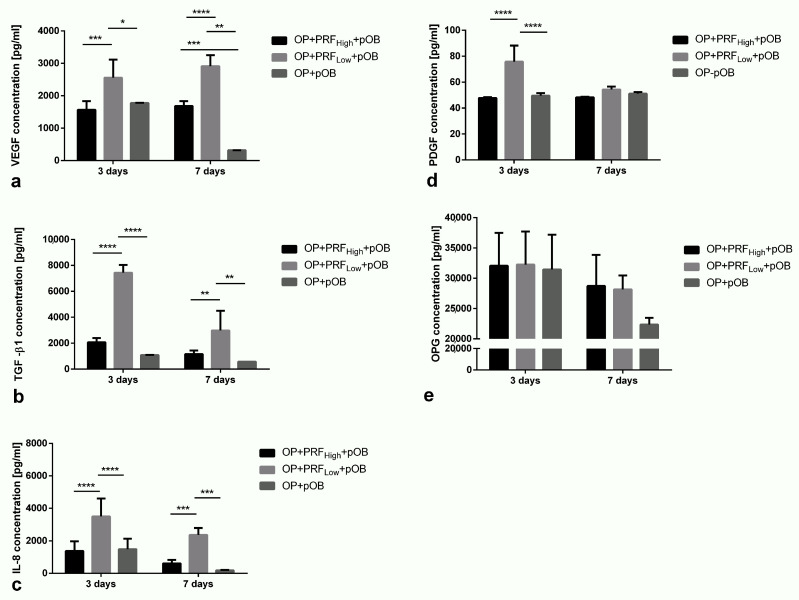
Evaluation of release of the growth factors (**a**) VEGF, (**b**) TGFβ1, (**c**) IL-8, (**d**) PDGF and (**e**) OPG, and in cell culture supernatants of pOBs cultivated on low and high RCF PRF coated Osteopore^TM^ meshes (OP+PRF_High_+pOB and OP+PRF_Low_+pOB) and compared to the release of growth factors when pOBs were cultivated on the pure Osteopore^TM^ meshes without PRF precoating (OP+pOB). *p*-value < 0.05 *, *p*-value < 0.01 **, *p*-value < 0.001 *** or *p*-value < 0.0001 ****.

The release of the growth factors VEGF, TGF β1, PDGF, OPG, and IL-8 was analyzed in cell culture supernatants of pOBs cultivated on low and high RCF PRF coated Osteopore^TM^ meshes (OP+PRF_High_+pOB and OP+PRF_Low_+pOB) and compared to the release of growth factors when pOBs were cultivated on the pure Osteopore^TM^ meshes without PRF precoating (OP+pOB) (Figure 5). In general, after three and seven days of cultivation, the group of OP+PRF_Low_+pOB reveal significantly higher concentration of IL-8, TGF-β1, and VEGF compared to the non-coated group and the group when high RCF PRF (OP+PRF_High_+pOB) was used as coating (Figure 5a–c). In addition, after three days of cultivation, the concentration of the analyzed growth factors VEGF, TGF-β1, and IL-8 in supernatants of pOBs cultivated on OP without PRF coating (OP+pOB) is comparable to the concentration of those factors in the high RCF PRF coated group (OP+PRF_High_+pOB). Remarkably, the release of VEGF, TGF-β1, and IL-8 in supernatants of cells cultivated on OP meshes without coating decreases significantly after seven days of cultivation whereas the growth factor content in pOBs on PRF coated materials remains on the same level for pOBs cultivated on high RCF PRF coated meshes. Concentration of VEGF in both PRF groups with cultivated pOBs remains stable up to seven days of cultivation (Figure 5a). Although TGF-β1 and IL-8 amount in supernatants of pOBs cultivated on either low or high RCF coated meshes also decreases during the course of cultivation, concentration can still be calculated on a higher level in comparison to the pOB on the uncoated materials (Figure 5b,c). Additionally, the highest amount of PDGF can be detected in OP+PRF_Low_+pOB supernatants after three days of cultivation, whereas the PDGF content in the other groups is on the same lower level (Figure 5d). After seven days of cultivation, PDGF concentration in OP+PRF_Low_+pOB decreases clearly. Furthermore, OPG concentration in cell culture supernatants reveals no differences between the experimental groups after three days of cultivation (Figure 5e). From three to seven days of cultivation, OPG content decreases in the OP+pOB group without additional PRF coating.

## 3. Discussion

This study analyzed the effect of biomaterial biologization of PCL mesh with platelet rich fibrin (PRF) for bone regeneration. Two different PRF matrices, generated according to the low speed centrifugation concept (LSCC), were applied in vitro to investigate their potential regenerative capacity for bone regeneration. Currently, the reconstruction and regeneration of large bone defects is one of the major challenges in different clinical fields, especially with regard to oral and maxillofacial surgery. In this context, inactive biomaterials often face limitations, and the need for autologous bone transplantation becomes mandatory to support bone regeneration [30]. The present study aimed to enhance the regenerative capacity of a PCL-mesh by means of biologization using different PRF-matrices.

PRF was introduced as a beneficial clinically applicable system that allows the use of a less invasive autologous basis for tissue regeneration. PRF can be generated from patients own peripheral blood by means of one step centrifugation [14]. Previously, introducing LSCC showed that reducing the applied RCF correlates with significantly higher number of blood cells and significantly higher bioactivity of PRF [18]. Based on these findings, the present study analyzed the combination of a PCL-mesh with liquid PRF matrices, centrifuged using either a high or a low RCF to outline whether biomaterial biologization by means of PRF-coating could be beneficial for bone regeneration and to identify the most suitable PRF preparation protocol for this indication. Primary human osteoblasts (pOBs) were used as a model to elucidate the potential effect on bone regeneration in vitro. To the best of our knowledge this study is the first to investigate the combination of different PRF matrices, as an autologous biologization component with PCL-mesh and pOBs in vitro.

The results showed that PRF-coating of a PCL-mesh (OP) allows its biologization as shown by histological staining. Both PRF matrices built a thin but stable fibrin matrix along the PCL-mesh surface and served as an additional natural autologous matrix for the pOBs. The PRF network was also observed in the interspace between the single mesh items in both groups. These findings confirm that the surface of the here analyzed PCL-meshes exhibits suitable physico-chemical properties that favored the adhesion of the PRF matrices to their surfaces. By contrast, a previous study showed that different biomaterials react differently, when combined with liquid PRF matrices [22]. Thereby, some collagen-based matrices exhibited a high potential to absorb liquid PRF, whereas others did not allow liquid PRF to penetrate into their matrix, although both materials were derived from the same source i.e., porcine [22]. Moreover, the combination OP+PRF_Low_ released significantly higher concentrations of growth factors (VEGF, TGF-β1, PDGF) as well as IL-8 compared to OP+PRF_High_, as demonstrated by ELISA measurements. These outcomes are supported by numerous previous studies that observed similar influence of the applied RCF on PRF bioactivity [18,19]. The here presented findings are most probably explained by the higher number of cells observed in the group of OP+PRF_Low_, that produced significantly higher growth factor concentrations compared to the low cells number observed in the group of OP+PRF_High._ Additionally, no negative effect of the combination of OP with the PRF matrices was observed in terms of enhanced inflammation or adverse cellular reaction.

In the present study, pOBs were seeded on the biologized PRF-coated meshes OP+PRF_High_ and OP+PRF_Low_.pOBs seeded on native OP were considered as a control group. The pOBs were allowed to adhere on the respective meshes for 24 h. Thereafter, the constructs (OP+PRF_High_+pOBs, OP+PRF_Low_+pOBs and OP+pOBs) were transferred to a new plate to eliminate artificial influence of cells that adhered to the well surface and focus on the pOBs adherent to the biomaterial. After three days and seven days, immunofluorescence staining showed that the highest number of adherent pOBs was found in the PRF-coated group of OP+PRF_Low_+pOBs compared to all other groups. Both PRF cells and pOBs were observed on the surface of OP+PRF_Low_+pOBs. Less pOBs were found in the group of OP+PRF_High_+pOBs. Notably, a comparably low number of pOBs were observed on the surface of uncoated native OP in the group OP+pOBs. In this case, a higher number of pOBs was detected on the well surface and less were adherent to OP itself. One limitation of the present study is the use of only one osteogenic marker. In this case, pOBs were assessed by DAPI and immunofluorescence staining of osteopontin, which is mostly expressed by differentiated osteoblasts. Further early osteogenic marker such as collagen, actin or calcein may have highlighted a clearer effect.

Previous studies have shown that osteoblasts adhesion is dependent on the introduced surface morphology [31]. Additionally, the role of fibrin as a useful matrix for cellular adhesion, migration, proliferation, and differentiation, especially for bone tissue engineering is well documented in different studies [32]. Previously, xenogeneic fibrin such as fibrinogen from bovine plasma was used in tissue engineering and showed promising results when combined with biomaterials and different cell types including stem cells [33] and osteoblasts [34]. Thereby, the present focus on the autologous fibrin in PRF. The results demonstrated that PRF served as an additional natural matrix for the pOBs and supported their adhesion on the OP surface. This effect was higher, when liquid PRF was centrifuged using a low RCF. Similar results were shown by a previous study, when osteoblasts were added on native titanium or titanium that was either coated with platelet-rich plasma or platelet-rich fibrin. In that study, cell adhesion was the highest in the PRF group, which again highlight the role of fibrin coating for cell adhesion [35]. In a previous study of our group, effect of injectable PRF has been analyzed on osteogenic differentiation capacity of a co-culture system of pOB and endothelial cells [21]. When pOB in monoculture were mixed with PRF and cultivated for seven days, osteopontin-positive cells clearly accumulated at sites of monocytic-like cells. Furthermore, a recent in vitro study analyzed the migration of zoledronic acid-treated osteoblasts, when additionally treated with PRF in vitro. The results demonstrated a significantly higher osteoblast migration after PRF treatment compared to the negative control [34].

Additionally, PRF-coated meshes activated pOBs to release significantly higher concentrations of different growth factors (VEGF, TGF-β1, and PDGF), IL-8 as well as alkaline phosphatase (ALP) activity compared to the uncoated meshes. Interestingly, on day 7, the highest ALP enzymatic activity was found in the group of OP+PRF_Low_+pOBs. This observation supports the hypothesis, that biologization of PCL-meshes by means of liquid PRF significantly enhance the differentiation of pOBs. The results are consistent with a previous study in which an upregulation of ALP expression could be documented when pOBs were cultivated with PRF [21]. ALP is known as a marker for differentiating osteoblasts and was previously shown to upregulate matrix mineralization in native bone [36]. The induction of ALP in human bone marrow stromal cells was shown to be associated with enhanced bone forming capacity after their implantation in vivo [37]. Consequently, the enhanced ALP activity in the group of OP+PRF_Low_+pOBs is a promising effect for bone regeneration. Moreover, the here detected growth factors are of great influence on bone regeneration. VEGF is one of the most important factors for angiogenesis [38,39], which is mandatory for bone regeneration, especially in large defects where lack of vascularization may lead to necrosis and consequently augmentation failure. The essential role of VEGF in angiogenesis was previously demonstrated using prefabricated scaffolds loaded with recombinant VEGF [40]. Similarly, liquid PRF offers an autologous and natural source of VEGF, as shown by the here presented results. The role of PRF as a natural scaffold and bioactive material for the support of new vessel formation by VEGF release was previously demonstrated in vitro [21]. In that study, a co-culture of pOBs and endothelial cells was embedded in the PRF matrix, which improved new vessel formation and led to the building of well-organized vessel network [21]. TGF-β1 was shown to contribute to angiogenesis [41] and to regulate bone remodeling [42,43]. Previous studies demonstrated the significant association between TGF-β1 levels, bone volume [43], and bone quality [42]. Moreover, the present study showed a significantly higher release of PDGF when pOBs were seeded on coated meshes, especially in the early time point of three days. PDGF is released from the alpha granules of platelets [44]. In addition to its role in neoangiogenesis and vessel stabilization [45,46,47], different studies demonstrated the effect of PDGF on new bone formation [44,48]. Although the detailed mechanism is yet unclear, PDGF was show to be involved in a multicomponent cascade influencing the vasculature-pericyte-osteoblast dynamics and thereby contribute to new bone formation [49]. Furthermore, a recent in vivo study analyzed the effect of PDGF loaded collagen matrix on bone formation and showed a significantly higher new bone formation induced by PDGF compared to the unloaded collagen matrix [48]. The results of IL-8 release showed significantly higher concentrations in the PRF-coated groups, especially OP+PRF_Low_+pOBs compared to uncoated OP. IL-8 is an inflammatory cytokine that recently gained importance in bone regeneration. It was reported to have a high recruitment efficiency for bone marrow stem cells and to be involved in chondrogenesis and osteogenesis in addition to its immune regulatory functions [50]. A previous study demonstrated the enhanced therapeutic effects of BMSCs on bone regeneration, when combined with IL-8 [51]. Moreover, IL-8 shows a regenerative effect by enhancing the osteoinductivity of BMP-2 leading to rapid initiation of guided bone regeneration [52]. Interestingly, the combination of IL-8 and BMP-2 demonstrated a synergistic effect of these signaling molecules and lead to enhanced regeneration of osteochondral defects in vivo [53]. The present results demonstrated additionally that the OPG release in the PRF-coated groups was higher compared to the uncoated group after seven days of cultivation. Although this can be observed as a clear trend, the difference was not statistically significant. The role of OPG in the regulation of bone homeostasis is well documented [54,55]. By binding to RANKL, OPG inhibits osteoclastogenesis and osteoclasts activity and allows the domination of osteoblasts [54]. Previous studies provided some insights about the effect of OPG/RANKL balance in guided bone regeneration and its potential role in preventing implant failures [56]. The here observed trend of a high growth factor release of different growth factors and cytokines in the group of OP+PRF_Low_+pOBs may be explained by the synergistic effect of cells included in PRF (Leukocytes and Platelets) as well as osteoblasts. Additionally, the fibrin network provides binding sites for different growth factors [39], that may have led to a continuous growth factor release especially on day 7. This positive effect is promising for supporting bone regeneration. Thereby, this combination built a complex biological system in which the different cells seem to interact with each other via cell–cell communication and various signaling molecules. Therefore, the present study failed to specifically elucidate the mechanism of this enhanced growth factor release in this complex system.

Altogether, the present study demonstrated that PCL mesh biologization by PRF-coating enhanced its biocompatibility and bioactivity. The proposed biologization protocol using PRF matrices provided a natural and bioactive scaffold for osteoblasts in vitro. The biologized PCL mesh, especially OP+PRF_Low_+pOBs enhanced the activity of pOBs to release different growth factors, essential for bone regeneration. The here presented results are of great clinical interest and may provide a clinically applicable tool to support bone regeneration and reconstruction as an alternative for autologous bone transplantation and might reduce patients’ morbidity. Further research, especially in vivo is needed to proof these findings.

## 4. Materials and Methods

Primary cells that were used for this study were obtained from excess tissue and their application was in accordance with the principle of informed consent and approved by the responsible Ethics Commission of the state of Hessen, Germany.

### 4.1. Osteopore^TM^ PCL Mesh

Osteopore^TM^ (OP) is a commercially available PCL mesh and was provided from Osteopore International Pte Ltd., Singapore. According to the manufacturer, this flexible biodegradable material is indicated to be use for cranial and facial reconstruction in craniomaxillofacial surgery. The PCL scaffold was fabricated using a Fused Deposition Modeling (FDM) technique to create unique honeycomb microarchitecture (Osteopore International Pte Ltd., Singapore). Briefly, alternating layers of PCL filaments were laid down in a pre-determined fashion of 0/60/120o.

### 4.2. Platelet Rich Fibrin Assembly and Characterization

The application of PRF in this study was approved by the responsible Ethics Commission of the state of Hessen, Germany (265/17). Peripheral blood was collected from 3 healthy donors using a vacuum blood collection butterfly from the antecubital vein and transferred directly into 10-mL i-PRF^®^ tubes (Process for PRF, Nice, France) and immediately centrifuged in a Duo^®^ centrifuge (Process for PRF, Nice, France). According to the Low-Speed Centrifugation Concept (LSCC), PRF was prepared using a high- (710× *g*) and a low-RCF (44× *g*) centrifugation protocol. The injectable PRF of the respective centrifugation protocol (PRF_High_ and PRF_Low_) was collected into a syringe, homogenized, and the resulting homogenized PRF was used for the biologization of the PCL meshes. Therefore, the PCL meshes were cut into pieces (7 mm × 7 mm) and transferred to a 24-well cell culture plate before 100 µL of the liquid PRF (PRF_High_ and PRF_Low_) was added to the material. After allowing clotting of the PRF via incubating at 37 °C for 20 min, 400 µL of cell culture medium Dulbecco’s Modified Eagle’s Medium (DMEM) was added and the different material-PRF-compositions were cultivated for 3 and for 7 days. Subsequently, the constructs were characterized morphologically via H&E staining as well as with regard to growth factor and cytokine release using ELISA.

### 4.3. Cell Culture Experiments

Human primary osteoblasts (pOB) were isolated from cancellous bone fragments from healthy donors according to an established protocol [57]. Isolated cells were cultivated in Dulbecco’s Modified Eagle Medium Nutrient Mixture F-12 (DMEM; Sigma-Aldrich, St. Louis, MO, USA) supplemented with 10% fetal bovine serum (FBS; Biochrom, Berlin, Germany) and 1% Penicillin streptomycin (P/S; Sigma-Aldrich, St. Louis, MO, USA) at 37 °C in a humidified atmosphere, were passaged in a ratio of 1:2 and were used up to passage 3 for this study. The polymeric resorbable scaffolds (Osteopore^TM^) were pre-coated with differently centrifuged platelet rich fibrin (PRF_High_ and PRF_Low_) before cell culture experimentation. For coating of the material, 100 µL of the respective liquid PRF was added to the material per well (24-well plates) and was incubated at 37 °C for 20 min to allow clotting before human primary osteoblasts (10,000 cells per well) were seeded on top of the material for 3 and 7 days of cultivation. To avoid cell adhesion on cell culture plastic, the PRF-coated scaffold with the pOBs seeded on top of the scaffolds were transferred to a new well after 24 h of cultivation to allow the evaluation of only adherent cells on the materials surface. Primary osteoblasts cultured on the material without PRF were used as controls. After 3 and 7 days of cultivation, supernatants were collected for growth factor and cytokine evaluation and cells were further processed for immunofluorescent, immunohistochemical staining, as well as for analyzation of enzymatic activity and cell viability.

### 4.4. Histochemistry

After 3 and 7 days of cultivation, samples were fixed in 4% buffered formalin (Roti-Histofix, Carl-Roth) and were dehydrated in an ascending ethanol series using a tissue processor (Leica TP1020, Wetzlar, Germany). Subsequently, samples were embedded in paraffin blocks to perform histological sections with a thickness of 3–4 µm generated using a microtome (Leica RM2255). One slide of each experimental PRF group was stained with hematoxylin and eosin (H&E) for general evaluation of cell distribution and general morphology. Additionally, immunohistochemical staining using the osteogeneic marker opsteopontin (ThermoFisher, Germany, RB-9097) for osteoblasts identification as previously described [21]. Stained samples were analyzed using a light microscope (Nikon, Tokyo, Japan) with a scanning table (Prior, Rockland, MA, USA) connected to a digital camera (DS-Fi/1, Nikon). Total scans were created using NIS Elements software (version 4.1, Nikon) by merging 50–100 single images of one sample.

### 4.5. Immunofluorescence Staining

For immunofluorescent staining, cell culture samples were fixed in 4% paraformaldehyde (ROTI^®^Histofix) for at least 5 min before washing with PBS and permeabilising with 0.01% Triton X/PBS. After washing again three times with PBS, cell cultures were incubated with mouse anti-human Osteopontin (1:100; Neomarkers Fremont Lot: 9097P10707A) diluted in a 1% bovine serum albumin/PBS solution for 60 min at room temperature. After washing three times with PBS, the cells were incubated with a red secondary anti-mouse antibody Alexa 546 (Molecular Probes, MoBiTec, Göttingen, Germany) diluted 1:1000 in a 1% bovine serum albumin/PBS solution for 60 min at room temperature, protected from light. The cells were additionally stained with Dapi to visualize cell nuclei and were mounted with Fluoroshield (ImmunoBioScience Corp., Mukilteo, WA, USA) and examined using a fluorescence microscope (Nikon eclipse TS100, Düsseldorf, Germany).

### 4.6. Enzyme-Linked Immunosorbent Assay (ELISA)

After 3 and 7 days of cultivation, cell culture supernatants of the pOBs cultivated on the PRF-coated scaffolds as well as control supernatants were collected and analyzed for amount of Vascular endothelial growth factor (VEGF), Transforming growth factor β1 (TGF β1), Platelet-derived growth factor (PDGF), Osteoprotegerin (OPG), and Interleukin 8 (IL-8). The concentration of the different proteins was measured using DuoSet^®^ ELISA Development Systems according to the manufacturer’s protocol (R&D Systems). A streptavidin-HRP colorimetric reaction was used to visualize protein concentrations and the optical density of each well was measured using a microplate reader (Tecan, Crailsheim) at a wavelength of 450 nm. Results are demonstrated as absolute values as indicated in the relevant figure.

### 4.7. Determination of Alkaline Phosphatase Enzymatic Activity

Quantitative determination of alkaline phosphatase (ALP) activity within cell culture supernatants was performed using p-nitrophenyl phosphate (pNPP) according to the manufacturer’s instructions (Alkaline phosphatase assay kit, Abcam, ab83369). Therefore, 50 µ of 5 mM pNPP solution was added to each well for 60 min at 25 °C protected from light. After the incubation period 20 µL of stop solution was transferred to each sample before absorbance was measured at 405 nm using a microplate reader (Tecan, Crailsheim, Germany). Alkaline phosphatase activity was calculated and displayed as relative quantification.

### 4.8. Statistical Analyses

All experiments were performed with at least 3 different donors. The data are presented as mean values ± standard deviation. Statistical significance was evaluated using one-way analysis of variance with Tukey’s multiple comparison test (α = 0.05). Statistical analyses were performed with Graph Pad Prism 8 (graph Pad Software, Inc, La Jolla, USA) and differences were considered significant when *p*-value < 0.05 *, *p*-value < 0.01 **, *p*-value < 0.001 *** or *p*-value < 0.0001 ****, respectively.

## 5. Conclusions

The present study introduced a biomaterial biologization system for bone regeneration. The here applied natural and autologous PRF, prepared according to LSCC, significantly enhanced the biocompatibility and bioactivity of PCL and thereby positively influenced osteoblasts activity in vitro. PRF is a clinically applicable system and may be considered as a less invasive natural source of patients own regenerative cells. The combination of PRF-coated PCL with osteoblasts demonstrated for the first time a promising regenerative effect of PRF, when combined with non-active biomaterials. Further in vivo and clinical studies are highly recommended to proof the potential therapeutic benefit.

## Data Availability

Not applicable.
